# Whole-body MRI versus an [^18^F]FDG-PET/CT-based reference standard for early response assessment and restaging of paediatric Hodgkin’s lymphoma: a prospective multicentre study

**DOI:** 10.1007/s00330-021-08026-1

**Published:** 2021-05-22

**Authors:** Suzanne Spijkers, Annemieke S. Littooij, Thomas C. Kwee, Nelleke Tolboom, Auke Beishuizen, Marrie C. A. Bruin, Goya Enríquez, Constantino Sábado, Elka Miller, Claudio Granata, Charlotte de Lange, Federico Verzegnassi, Bart de Keizer, Rutger A. J. Nievelstein

**Affiliations:** 1 Department of Radiology and Nuclear Medicine, University Medical Centre Utrecht/Wilhelmina Children’s Hospital, Utrecht University, Heidelberglaan 100, 3584 CX Utrecht, The Netherlands; 2 Princess Máxima Centre for Paediatric Oncology, Utrecht, The Netherlands; 3 Department of Radiology, Medical Imaging Centre, University Medical Centre Groningen, University of Groningen, Groningen, The Netherlands; 4 Department of Paediatric Oncology/Haematology, Erasmus Medical Centre-Sophia Children’s Hospital, Rotterdam, The Netherlands; 5 Department of Pediatric Radiology, University Hospital Vall d’Hebron, Institut de Recerca Vall d’Hebron, Barcelona, Spain; 6 Department of Paediatric Oncology and Haematology, University Hospital Vall d’Hebron, Barcelona, Spain; 7 Department of Medical Imaging, CHEO, University of Ottawa, Ottawa, Canada; 8 Department of Radiology, Institute for Maternal and Child Health IRCCS Burlo Garofolo, Trieste, Italy; 9 Department of Diagnostic Imaging and Intervention, Oslo University Hospital, Rikshospitalet, Oslo, Norway; 10 Oncohematology Unit, Institute for Maternal and Child Health IRCCS Burlo Garofolo, Trieste, Italy

**Keywords:** Whole-body imaging, Child, Diffusion magnetic resonance imaging, Hodgkin disease, Positron emission tomography computed tomography, observer variation

## Abstract

**Objectives:**

To compare WB-MRI with an [^18^F]FDG-PET/CT-based reference for early response assessment and restaging in children with Hodgkin’s lymphoma (HL).

**Methods:**

Fifty-one children (ages 10–17) with HL were included in this prospective, multicentre study. All participants underwent WB-MRI and [^18^F]FDG-PET/CT at early response assessment. Thirteen of the 51 patients also underwent both WB-MRI and [^18^F]FDG-PET/CT at restaging. Two radiologists independently evaluated all WB-MR images in two separate readings: without and with DWI. The [^18^F]FDG-PET/CT examinations were evaluated by a nuclear medicine physician. An expert panel assessed all discrepancies between WB-MRI and [^18^F]FDG-PET/CT to derive the [^18^F]FDG-PET/CT-based reference standard. Inter-observer agreement for WB-MRI was calculated using kappa statistics. Concordance, PPV, NPV, sensitivity and specificity for a correct assessment of the response between WB-MRI and the reference standard were calculated for both nodal and extra-nodal disease presence and total response evaluation.

**Results:**

Inter-observer agreement of WB-MRI including DWI between both readers was moderate (*κ* 0.46–0.60). For early response assessment, WB-MRI DWI agreed with the reference standard in 33/51 patients (65%, 95% CI 51–77%) versus 15/51 (29%, 95% CI 19–43%) for WB-MRI without DWI. For restaging, WB-MRI including DWI agreed with the reference standard in 9/13 patients (69%, 95% CI 42–87%) versus 5/13 patients (38%, 95% CI 18–64%) for WB-MRI without DWI.

**Conclusions:**

The addition of DWI to the WB-MRI protocol in early response assessment and restaging of paediatric HL improved agreement with the [^18^F]FDG-PET/CT-based reference standard. However, WB-MRI remained discordant in 30% of the patients compared to standard imaging for assessing residual disease presence.

**Key Points:**

*• Inter-observer agreement of WB-MRI including DWI between both readers was moderate for (early) response assessment of paediatric Hodgkin’s lymphoma.*

*• The addition of DWI to the WB-MRI protocol in early response assessment and restaging of paediatric Hodgkin’s lymphoma improved agreement with the [18F]FDG-PET/CT-based reference standard.*

*• WB-MRI including DWI agreed with the reference standard in respectively 65% and 69% of the patients for early response assessment and restaging.*

**Supplementary Information:**

The online version contains supplementary material available at 10.1007/s00330-021-08026-1.

## Introduction

Paediatric Hodgkin’s lymphoma is nowadays a highly curable malignancy and it is the most common type of cancer in adolescents [1, 2]. Accurate assessment of response therapy, both after the first two courses of chemotherapy (early response assessment, ERA) and at the end of therapy (restaging), is of great importance for outcome prediction and tailoring the therapy schedule to the individual patient. 2-[^18^F]fluoro-2-deoxy-D-glucose positron emission tomography/computed tomography ([^18^F]FDG-PET/CT) provides not only anatomical but also functional metabolic information. [^18^F]FDG-PET/CT is the current recommended imaging modality in international guidelines for (interim) response assessment [3].

A drawback of [^18^F]FDG-PET/CT is the administration of ionizing radiation, especially if a high-dose rather than a low-dose CT is used [4]. Moreover, in many hospitals around the world, a contrast-enhanced CT is still part of standard clinical procedures [4]. In between the initial diagnosis and end of treatment, children diagnosed with Hodgkin’s lymphoma undergo multiple imaging examinations and are thus exposed to a substantial dose of ionizing radiation. It is shown in the literature that children as compared to adults have an increased vulnerability to the long-term side effects of ionizing radiation such as secondary malignancies [5–8]. This underlines the need for careful administration of ionizing radiation in childhood, both for therapy purposes and in diagnostic imaging protocols.

Whole-body magnetic resonance imaging (WB-MRI) with diffusion-weighted imaging (DWI) was already shown to be a radiation-free alternative with high accuracy for staging paediatric Hodgkin’s lymphoma [9–13]. The functional information provided by DWI has been found to be a useful addition to conventional WB-MRI protocols for staging Hodgkin’s lymphoma [13], but despite these positive results for staging, the first studies focussing on response assessment do not show consistent results. Some authors have reported that WB-MRI cannot match [^18^F]FDG -PET/CT for response assessment [10, 14–16], while others report that WB-MRI may be a useful alternative not only in staging but also in treatment response assessment in lymphoma patients [16–19]. Since most of these studies focus on heterogeneous lymphoma populations with different tumour characteristics and treatment protocols, drawing conclusions on the value of WB-MRI in paediatric Hodgkin’s lymphoma is challenging [20].

Therefore, the aim of this prospective, multicentre study was to assess the performance of WB-MRI as compared to an [^18^F]FDG-PET/CT-based reference standard in the detection of residual disease in early response assessment and restaging after completion of therapy in a study population comprising only paediatric Hodgkin’s lymphoma patients.

## Methods

For this prospective international multicentre cohort study, patients were recruited in nine hospitals: University Medical Centre Utrecht, University Children’s Hospital Vall d’Hebron Barcelona, Amsterdam University Medical Centres, CHEO-Ottawa, Giannina Gaslini Children’s Hospital Genova, Erasmus Medical Centre – Sophia Children’s Hospital Rotterdam, Materno Infantile Burlo Garofolo Trieste and Oslo University Hospital Rikhospitalet. All institutional review boards provided approval of this prospective study. Written informed consent was obtained from all study participants and/or their parents or legal guardians.

### Patient population

The patients included from the European participating hospitals also participated in the EuroNet PHL-C1 trial (First international Inter-Group Study for classical Hodgkin’s Lymphoma in Children and Adolescents) [21, 22]. Inclusion criteria for the current study were children aged 10–17 years old, with histologically proven Hodgkin’s lymphoma. Exclusion criteria included general MRI contraindications such as pacemakers and claustrophobia. Patients were also excluded if the number of days between [^18^F]FDG-PET/CT and WB-MRI exceeded 15 days for ERA or 30 days for restaging. All patients were included between 2012 and 2016 and staging results of the participants were reported in the previous article [13].

### WB-MRI and [^18^F]FDG-PET/CT procedures

All patients underwent both WB-MRI and [^18^F]FDG-PET/CT examinations after two cycles of chemotherapy (ERA, performed 14–17 days after chemotherapy) (Table 1). A subset of patients also received both imaging examinations after completion of therapy (restaging) (performed 14–17 days after completion of therapy). WB-MRI and [^18^F]FDG-PET/CT were performed within a 15-day timeframe for ERA (median 0.0 days, interquartile range (IQR) −4 to 0.5) and a 30-day timeframe for restaging after therapy (median 0.0 days, IQR −1 to 3.5).

**Table 1:** Patient characteristics

	Early response assessment *N* (%) *n* = 51	Restaging *N* (%) *n =* 13
Age (years)		
– Mean (standard deviation) – Range	14.1 (2.4) 10-17	14.2 (2.1) 10-17
Gender		
– Male – Female	24 (47) 27 (53)	9 (69) 4 (31)
Hodgkin’s lymphoma subtype		
Classical		
◦ Nodular sclerosing HL ◦ HL, lymphocyte rich ◦ HL, mixed cellularity ◦ Classical HL, not otherwise specified	28 (55) 4 (8) 4 (8) 15 (29)	5 (39) 0 (-) 1 (8) 7 (54)
Initial stage		
– I/II (limited disease) – III/IV (advanced disease)	38 13	0 13
Response		
– Complete response (CR) – Partial response (PR) – Stable disease (SD) – Progressive disease (PD)	38 (75) 13 (25) 0 (-) 0 (-)	11 (85) 1 (8) 0 (-) 1 (8)
Therapy before ERA		
– 2 cycles ABVD	1 (2)	
– 2 cycles OEPA	45 (88)	
– 2 cycles OPPA	5 (10)	
Therapy following ERA		
– 2 cycles COPDAC		5 (39)
– 2 cycles COPDAC + RT		3 (23)
– 4 cycles COPDAC		2 (15)
– 4 cycles COPDAC + RT		1 (8)
– 4 cycles COPDAC + RT, 2 cycles IEP/ABVD + stem cell transplant + RT + Brentuximab		1 (8)
– 4 cycles DECOPDAC		1 (8)
Number of patients included per centre		
– University Medical Centre Utrecht	12 (24)	7 (54)
– University Children’s Hospital Vall d’Hebron Barcelona	14 (27)	
– Amsterdam University Medical Centres	7 (14)	4 (30)
– CHEO-Ottawa	8 (16)	
– Giannina Gaslini Children's Hospital Genova	2 (4)	
– Erasmus Medical Centre – Sophia Children’s Hospital Rotterdam	4 (8)	1 (8)
– Materno Infantile Burlo Garofolo Trieste	1 (2)	1 (8)
– Oslo University Hospital Rikhospitalet	3 (6)	

*ABVD*, Adriamycin (doxorubicin), bleomycin, vinblastine, dacarbazine; *COPDAC*, cyclophosphamide, doxorubicin, prednisone, dacarbazine; *DECOPDAC*, dacarbazine, etoposide, doxorubicin, cyclophosphamide, vincristine, prednisone; *ERA*, early response assessment; *HL*, Hodgkin’s lymphoma; *IEP*, ifosfamide, etoposide, prednisone; *OEPA*, vincristine, etoposide, prednisone, Adriamycin (doxorubicin); *OPPA*, vincristine, procarbazine, prednisone, Adriamycine; *RT*, radiotherapy

WB-MRI was performed using a 1.5-T system (Philips Healthcare) or Siemens or GE Medical Systems) or a 3.0-T system (Siemens) and image acquisition took place from the top of the head to the upper thigh. Coronal whole-body T1-weighted (T1W, except for CHEO, where only T2-weighted and diffusion-weighted images were acquired), T2-weighted images (T2W) and diffusion-weighted images (b0, b100 and b800 s/mm^2^) were acquired under free-breathing, except for the stations covering the chest and abdomen, which were acquired using breath-holding (T1W) or respiratory triggering (T2W). Seamless coronal whole-body T1W and T2W images were created by merging separately acquired stations using software implemented in the standard operating console. Axial diffusion-weighted images were first coronally reformatted and then merged to create seamless coronal whole-body diffusion-weighted images. The duration of the examination, including patient preparation time, was approximately 60 min. The detailed WB-MRI parameters were as described before [13] and are shown in the supplementary material file.

Approximately 60 min after the administration of [^18^F]FDG, PET images were acquired according to the European Association of Nuclear Medicine (EANM) recommendations [23]. PET images were combined with low-dose CT (Biograph 16 PET-CT or Biograph 40 Truepoint PET-CT, Siemens Healthcare; Gemini TOF PET-CT or Allegro, Philips Healthcare). Imaging was performed from mid-thigh to skull base.

### WB-MRI and [^18^F]FDG-PET/CT interpretation

After completion of all examinations, the images were collected and de-identified and distributed to the readers. The WB-MRI scans were reviewed by two independent radiologists (R.A.J.N. and T.C.K. with 25 and 10 years of MRI experience in (paediatric) oncology, respectively) and the [^18^F]FDG-PET/CT images were scored by a nuclear medicine physician (B.d.K. with 15 years of [^18^F]FDG-PET/CT experience in (paediatric) oncology). None of the readers had access to information regarding clinical status and other imaging or laboratory findings except from the initial staging scans, to be able to distinguish pre-existent lesions from new disease presence. Either OsiriX Lite Medical Imaging Software (Pixmeo, www.osirix-viewer.com) or Horos Project Software (www.horosproject.org) was used.

For scoring, all readers used a standardised form that was based on the EuroNet PHL C1 trial [21, 22]. The EuroNet PHL C1 trial is an international, multicentre, randomised controlled trial with the aims to reduce the use of radiotherapy in paediatric Hodgkin lymphoma and to compare different therapy strategies in patients with advanced or intermediate disease.

Residual disease presence was scored positive or negative for the predefined anatomical sites (10 nodal and all extra-nodal stations). Nodal stations were cervical, axillary, infraclavicular, mediastinal, pulmonary hilar, spleen, para-aortic, mesenteric, para-iliac and inguinal. The lymph node stations were considered negative for residual disease if all nodal lesions had regressed to ≤ 15 mm in the longest diameter [3]. The extra-nodal stations were considered negative for disease presence if no abnormal signal intensities or masses were found. Finally, response was reported as complete response (CR), partial response (PR), stable disease (SD) or progressive disease (PD) based on tumour diameters and [^18^F]FDG uptake as defined by the EuroNet PHL C1 protocol [3, 24]. For [^18^F]FDG-PET/CT, the Deauville scoring system was used. This is a 5-point score, based on visual assessment of the [^18^F]FDG uptake. At DWI, the presence of restricted diffusion was visually examined based on a high signal at high *b*-value DWI and a lower signal compared to muscles or the spinal cord at ADC [24].

The WB-MRI readers evaluated the images in two sets. First are the conventional sequences alone (T1-weighted and T2-weighted images) followed by the conventional sequences combined with DWI immediately thereafter. An independent paediatric radiologist (A.S.L., with 15 years of MRI experience in (paediatric) oncology) solved all discrepancies between both WB-MRI reviewers to form the consensus MRI datasets.

For [^18^F]FDG-PET/CT, residual lesions were considered positive if their activity exceeded the uptake in the mediastinal blood pool [3]. The criteria for treatment response were based on those used in the EuroNet PHL C1 trial [24] in which the mediastinal blood pool was still used as reference. It should be noted that in newer versions of the EuroNet trial the uptake in the liver is used as a reference. For this current study, however, new lesions with an uptake above the mediastinal blood pool were also considered positive if no other explanation (e.g. inflammation) was more likely to be the underlying cause of the lesion.

### Reference standard and intrinsic WB-MRI

All discrepancies between the scoring results from consensus WB-MRI including DWI and [^18^F]FDG-PET/CT were discussed by an independent expert panel consisting of a nuclear medicine physician (N.T., with 9 years of [^18^F]FDG-PET/CT experience in (paediatric) oncology) and a paediatric radiologist (A.S.L., with 15 years of MRI experience in (paediatric) oncology). The expert panel had access to all available data, including the results from clinical, histopathological and imaging examinations. For all discrepancies between WB-MRI including DWI and [^18^F]FDG-PET/CT, the expert panel decided whether the discrepancy was a reader error or an intrinsic error. Intrinsic errors were caused by the limitations of the imaging modality itself (e.g. caused by artefacts). Reader errors could either be an interpretation error (incorrect classification of an abnormality) or a perceptual error (reader did not detect the abnormality). To form the [^18^F]FDG-PET/CT-based reference standard, the expert panel corrected all reader and intrinsic errors from the [^18^F]FDG-PET/CT reading. The intrinsic WB-MRI was formed by removing all reader errors from the consensus WB-MRI including DWI.

### Statistical analysis

Inter-observer analyses were performed between both WB-MRI readers by calculating observed agreement and Cohen’s kappa values, which were interpreted as poor (*κ* < 0.2), fair (*κ* 0.2–0.4), moderate (*κ* > 0.4–0.6), good (*κ* > 0.6–0.8) and excellent (*κ* > 0.8) [25].

Agreement between WB-MRI without DWI, WB-MRI with DWI and intrinsic WB-MRI and the [^18^F]FDG-PET/CT-based reference standard was assessed by calculating total agreement, positive predictive value (PPV) and negative predictive value (NPV). Those were calculated between WB-MRI and the reference standard for lymphoma detection per patient (response classification). Sensitivity and specificity with corresponding 95% confidence intervals (CI) of WB-MRI without DWI, WB-MRI with DWI and intrinsic MRI for early response assessment and restaging were calculated against the reference standard. For the presence/absence of residual disease in the combined nodal and extra-nodal stations, true-positive (TP), false-positive (FP), false-negative (FN) and true-negative (TN) rates were calculated alongside the observed agreement.

For all analyses in which multiple stations were assessed together, clustering within patients had to be considered. Multilevel analyses were performed as proposed by Vanbelle et al for the kappa statistics [26]. For observed agreement, PPV and NPV a mixed effect logistic regression model was used, taking clustering within patients into account using random intercepts.

All statistical analyses were performed using the Statistical Package for the Social Sciences (SPSS version 25.0,) or the R statistical software package version 3.5.1 (R development core team).

## Results

### Patient population

**Fig. 1:**
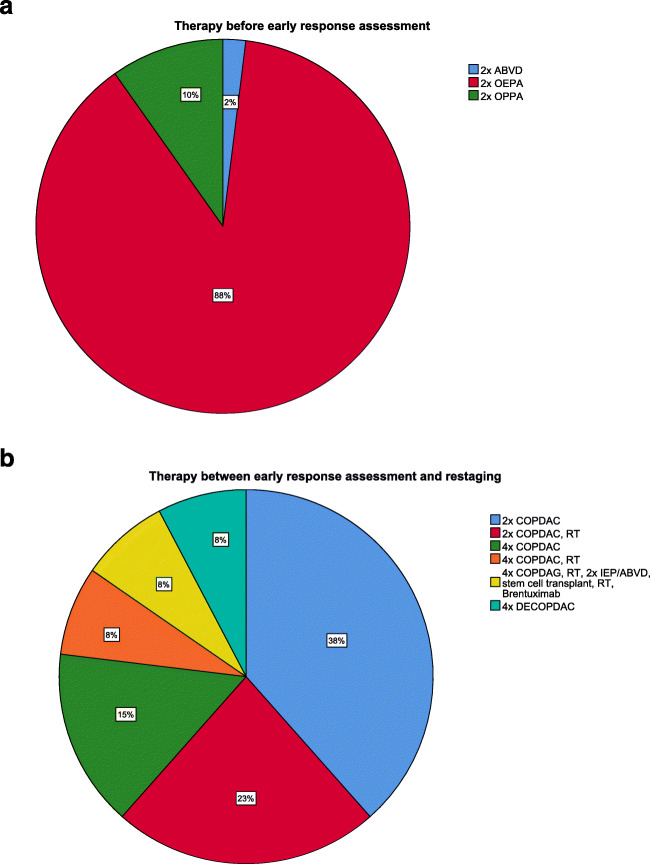
Pie chart showing the administered drug combinations. **a** Before early response assessment, **b** between early response assessment and restaging. ABVD, Adriamycin (doxorubicin), bleomycin, vinblastine, dacarbazine; COPDAC, cyclophosphamide, doxorubicin, prednisone, dacarbazine; DECOPDAC, dacarbazine, etoposide, doxorubicin, cyclophosphamide, vincristine, prednisone; ERA, early response assessment; HL, Hodgkin’s lymphoma; IEP, ifosfamide, etoposide, prednisone; OEPA, vincristine, etoposide, prednisone, Adriamycin (doxorubicin); OPPA, vincristine, procarbazine, prednisone, Adriamycine; RT, radiotherapy

Patient selection and inclusion were prospectively conducted between 2012 and 2016. A total of 54 patients were found eligible for inclusion. Three patients had to be excluded due to the following reasons: one patient had an incomplete MRI study, and in two patients, the time interval between WB-MRI and [^18^F]FDG-PET/CT exceeded 15 days for early response assessment. All remaining 51 patients underwent WB-MRI and [^18^F]FDG-PET/CT for early response assessment. A subset of 13 patients received both WB-MRI and [^18^F]FDG-PET/CT at restaging. Table 1 shows the patient characteristics, including age, gender, Hodgkin’s lymphoma subtype, response classification, received therapy and number of patients included per participating centre. The majority of the patients (88%) received two cycles of OEPA (vincristine, etoposide, prednisone, Adriamycin (doxorubicin)) before early response assessment and most patients (39%) received 2 cycles of COPDAC (cyclophosphamide, doxorubicin, prednisone, dacarbazine) between early response assessment and restaging. Figure 1 shows a pie chart of the administered drug combinations, both before early response assessment and between early response assessment and restaging.

### Inter-observer agreement WB-MRI including DWI

The inter-observer agreement between both WB-MRI readers is shown in Table 2 for early response assessment and restaging combined. The observed agreement for response classification was 73% (47/64, 62–83%) and Cohen’s kappa agreement was moderate (*κ* 0.74). Agreement for both nodal and extra-nodal disease involvement was 97%, with moderate kappa values (*κ* 0.47 for the nodal stations and *κ* 0.60 for the extra-nodal stations).

**Table 2:** Inter-observer agreement between WB-MRI (including DWI) readers: early response assessment and restaging combined

	Early response assessment + restaging (*n* = 64)	
	Observed agreement (total) (*n*/*n*, 95% CI)	Kappa (95% CI)
Response	0.73 (47/64, 0.62–0.83)	0.46 (0.28–0.65)
All nodal sites combined	0.97 (607/640, 0.94–0.98)	0.47 (0.29–0.65)
All extra-nodal sites combined	0.97 (187/192, 0.94–0.99)	0.60 (0.28–0.92)
All sites combined	0.97 (794/832, 0.94–0.98)	0.49 (0.33–0.65)

*CI*, confidence interval

### Expert panel: formation of reference standard and intrinsic WB-MRI

To form the [^18^F]FDG-PET/CT-based reference standard and the intrinsic WB-MRI including DWI dataset, the expert panel assessed all discrepancies between WB-MRI and [^18^F]FDG-PET/CT. A total of 39 discrepant disease sites were identified (4.7% of all disease sites, 32 for early response assessment and 7 for restaging) in 21 patients. Three reader errors in two patients were corrected for the [^18^F]FDG-PET/CT reading (one perception error and two interpretation errors). No intrinsic [^18^F]FDG-PET/CT errors were found. [^18^F]FDG-PET/CT artefacts were seen in a small minority of patients (< 5%) and were all related to brown fat activation. For the formation of the intrinsic WB-MRI dataset the expert panel corrected three reader errors, all of the perception errors. The errors were found in three different patients and in three different stations (cervical, mediastinal and spleen). The remaining 33 discrepancies were all intrinsic WB-MRI errors. In the vast majority (26/33, 79%), the discrepancy was caused by a residual lesion that exceeded size limits for MRI but was not metabolic active on [^18^F]FDG-PET/CT.

### Early response assessment

**Fig. 2:**
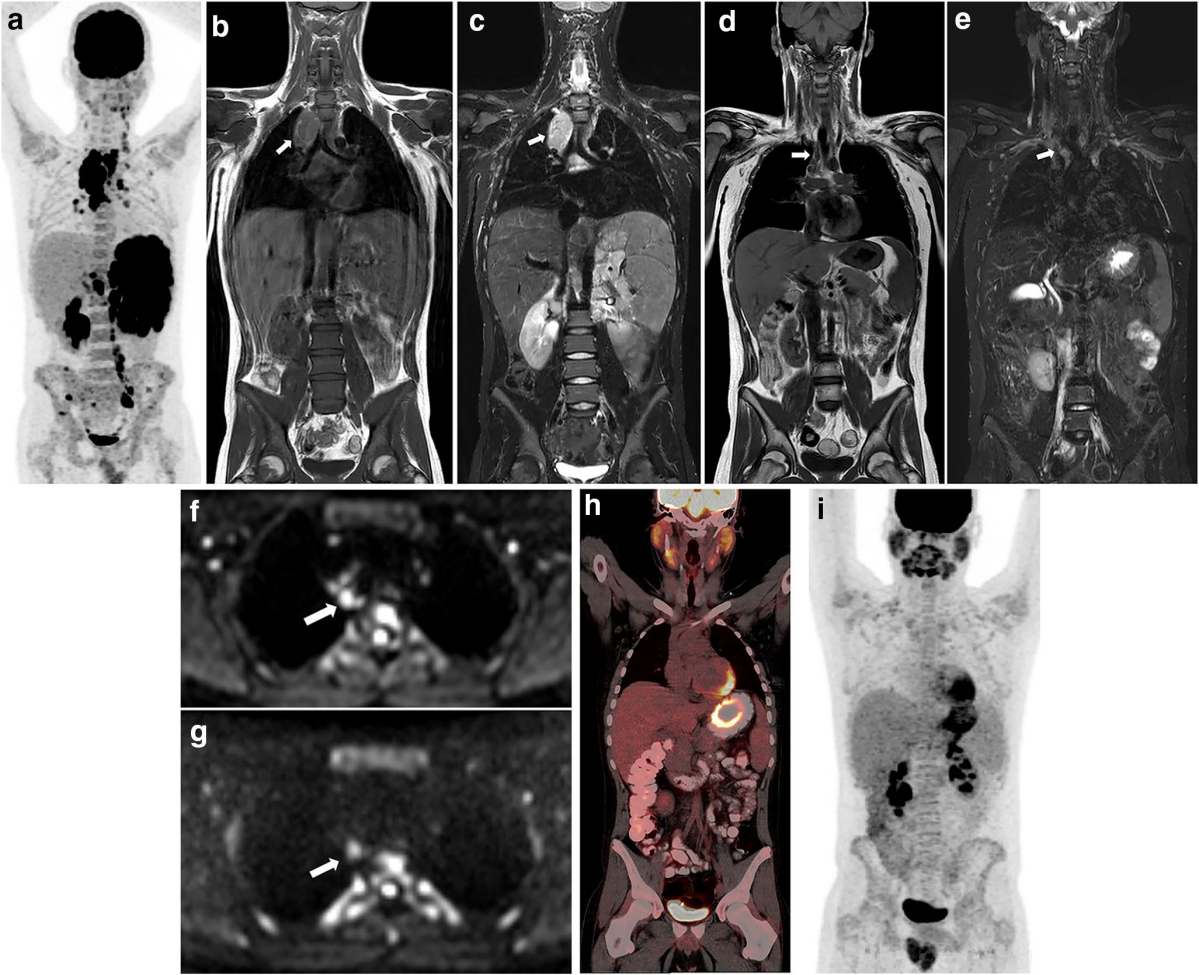
Example of an intrinsic WB-MRI error in early response assessment (ERA) after two cycles of chemotherapy. A 13-year-old boy initially diagnosed with stage III Hodgkin’s lymphoma. Maximum intensity projection (MIP) of the [^18^F]FDG-PET/CT at staging (**a**) shows several affected lymph node stations, including the spleen. T1-weighted (**b**) and T2-weighted (**c**) MRI at staging involvement of the mediastinum (arrows) was found. At ERA, coronal T1-weighted MRI (**d**) and T2-weighted MRI (**e**) show the mediastinal residual lesion (arrows). At axial DWI (b100 (**f**), b800 (**g**)), restricted diffusion was seen; the mediastinal station was therefore scored positive for disease presence (arrows). At coronal [^18^F]FDG-PET/CT (**h**), no elevated [^18^F]FDG uptake was seen in de mediastinum. Thus, the mediastinal lesion was scored positive for residual disease at ERA WB-MRI whereas [^18^F]FDG-PET/CT showed complete response. The [^18^F]FDG-PET/CT MIP (**i**) shows no elevated [^18^F]FDG uptake throughout the body, indicating a complete response

The diagnostic value of WB-MRI without DWI, WB-MRI with DWI and intrinsic WB-MRI for early response assessment is presented in Table 3. At WB-MRI without DWI, a total of 47 out of 51 patients were diagnosed with a residual lesion, but in only 12 out of those 47 patients, the reference standard was positive for residual lesions. The overall observed agreement between WB-MRI without DWI and the reference standard was 29%. Sensitivity and specificity for determining response status were 92% and 8% respectively for WB-MRI without DWI. The addition of DWI improved overall agreement (65%) and specificity (68%), but sensitivity decreased to 54%. For the intrinsic WB-MRI, the observed agreement was 71% (36/51, 95% CI 57-81%). Sensitivity improved (77%) and specificity remained the same (68%) compared to the WB-MRI reading. For all WB-MRI datasets, the NPV exceeded the PPV (ranging from 26 to 45%), but none exceeded 90%. Table 4 shows the amount of true-positive, false-positive, false-negative and true-negative disease stations and observed agreement for WB-MRI compared to the reference standard. Observed agreements for both nodal and extra-nodal residual disease detection for early response assessment are both 96%. More false-positive stations than false-negative stations are observed, predominantly for nodal disease (*n* = 19). In Fig. 2, an example of an intrinsic WB-MRI error in early response assessment is shown.

**Table 3:** Diagnostic value of WB-MRI versus the [^18^F]FDG-PET/CT-based reference standard for the early response assessment and restaging of paediatric Hodgkin’s lymphoma

	Observed agreement (95% CI)	Sensitivity (95% CI)	Specificity (95% CI)	PPV (95% CI)	NPV (95% CI)
Early response assessment					
MRI - DWI	29% (15/51, 19–43%)	92% (64–100%)	8% (2–21%)	26% (14–40%)	75% (19–99%)
MRI + DWI	65% (33/51, 51–76%)	54% (25–81%)	68% (51–82%)	37% (16–62%)	81% (64–93%)
Intrinsic MRI*	71% (36/51, 57–81%)	77% (46–95%)	68% (51–82%)	45% (24–68%)	90% (73–98%)
Restaging					
MRI - DWI	38% (5/13, 18–64%)	NA	45% (17–77%)	NA	83% (36–100%)
MRI + DWI	69% (9/13, 42–87%)	NA	82% (48–98%)	NA	90% (55–100%)
Intrinsic MRI*	69% (9/13, 42–87%)	NA	82% (48–98%)	NA	90% (55–100%)

*CI*, confidence interval; *DWI*, diffusion-weighted imaging; *MRI*, magnetic resonance imaging; *NA*, not applicable, insufficient numbers to perform analysis; *PPV*, positive predictive value; *NPV*, negative predictive value. *Intrinsic MRI, WB-MRI DWI after removal of reader error

**Table 4:** Nodal and extra-nodal response assessment: true-positive (TP), false-positive (FP), false-negative (FN) and true-negative (TN) disease stations and observed agreement for WB-MRI including DWI compared to the reference standard

	TP	FP	FN	TN	Observed agreement (95% CI)
Early response assessment					
Nodal	11	19	2	478	96% (94–97%)
Extra-nodal	2	4	2	145	96% (92–98%)
Restaging					
Nodal	1	2	2	125	97% (92–99%)
Extra-nodal	0	0	0	39	100% (97–100%)

*CI*, confidence interval

### Restaging after completion of therapy

**Fig. 3:**
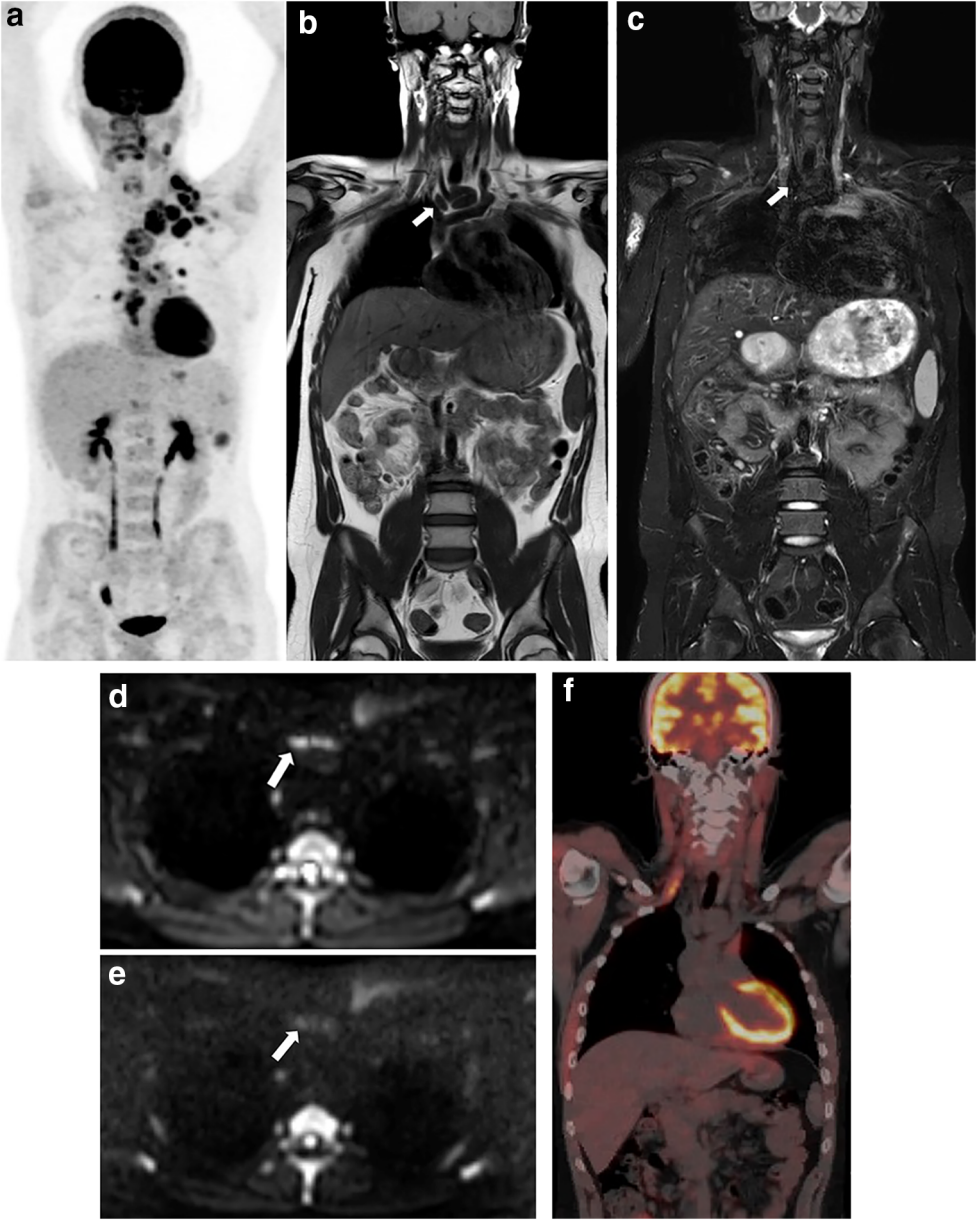
Example of end of treatment response evaluation (restaging) of a 15-year-old boy showing a fibrotic rest lesion without diffusion restriction at DWI and no disease presence at [^18^F]FDG-PET/CT. At staging, disease presence was found in the cervical, mediastinal, pulmonary hilar and para-aortic lymph node stations as well as in the spleen and right lung, indicating stage IV disease. Coronal maximum intensity projection (MIP) of the [^18^F]FDG-PET/CT (**a**) at staging demonstrates the affected (lymph node) stations. Coronal T1-weighted (**b**) and T2-weighted (**c**) MRI show a rest lesion at restaging (arrows). At both T1-weighted and T2-weighted MRI, low signal intensity is seen, and axial DWI (b100 (**d**) and b800 (**e**)) reveals no diffusion restriction. At axial [^18^F]FDG-PET/CT (**f**)**,** no elevated [^18^F]FDG uptake is seen in the rest lesion as well indicating a complete response

Only 13 patients were available for the restaging analysis. According to the reference standard, residual disease was present in one patient and one patient was diagnosed with progressive disease. Table 3 shows observed agreement, sensitivity, specificity and both PPV and NVP for restaging. The observed agreement between WB-MRI and the reference standard was 38% (5/13, 95% CI 18–64%) and improved with the addition of DWI to 69% (9/13, 95% CI 42–87%). In Table 4, the amount of true-positive, false-positive, false-negative and true-negative disease stations and observed agreement for WB-MRI including DWI compared to the reference standard are presented. Observed agreements for both nodal and extra-nodal residual disease detection for restaging are both high (97% and 100% respectively). Figure 3 shows an example of response evaluation at restaging.

## Discussion

This multicentre, prospective, international study compared WB-MRI with an [^18^F]FDG-PET/CT-based reference standard for detecting residual disease in early response assessment and restaging of paediatric Hodgkin’s lymphoma.

Results demonstrate a moderate inter-observer agreement between WB-MRI readers. This is comparable to a previous study in which inter-observer agreement for WB-MRI including DWI was moderate for restaging after treatment as well [14]. For extra-nodal disease, it should be noted that the relatively low kappa values are caused by a very low amount of stations in which extra-nodal residual disease involvement was found. The observed agreement for detecting extra-nodal disease between both WB-MRI readers was 98%, however, which is probably more indicative of the agreement between both readers.

For the per-lesion analyses, high percentages of observed agreement between [^18^F]FDG-PET/CT and WB-MRI including DWI (96–100%) were found for both nodal and extra-nodal detection of residual disease and for both early response assessment and restaging. However, especially for early response assessment, many false-positive stations were observed (around 4% of the total number of stations) compared to the reference standard. This indicates a low specificity for detecting nodal residual disease. This considerable number of false positives was in line with the expectations since it is known that residual masses are often detected without harbouring residual disease [27–29]. The additional value of DWI in detecting active disease was therefore analysed. With regard to the per patient analyses, the agreement between WB-MRI and the [^18^F]FDG-PET/CT-based reference standard improved with the addition of DWI for both early response assessment and restaging, but despite this major improvement, the agreements all remained insufficient with a maximum of 71% for early response assessment intrinsic WB-MRI. Similar conclusions can be drawn with regard to both sensitivity and specificity for detecting residual disease. This might be partly due to the amount of reader errors in the WB-MRI including DWI reading, as sensitivity and specificity both increased for the intrinsic WB-MRI, and also for the intrinsic WB-MRI data sensitivity and specificity remained inadequate (sensitivity 77% and specificity 68%).

This summary of our results points out that the value of WB-MRI for early response assessment and restaging is limited and cannot replace [^18^F]FDG-PET/CT, mainly due to an underestimation of response to treatment. These results are comparable to recent literature in which the value of WB-MRI during early response assessment and/or restaging in all types of lymphoma was questioned [10, 14, 15]. However, the results of the current study are opposite to the results from Herrmann et al, Maggialetti et al and Mayerhoefer et al, who all reported that WB-MRI might be a useful alternative in response assessment of lymphomas [16–18]. These different outcomes might be due to differences in study populations including different lymphoma types. The lymphomas comprise a heterogeneous group of tumours with different histological characteristics and treatment protocols. For the present study, only Hodgkin’s lymphoma patients were included, and therefore, our results might not be comparable to those earlier studies. [^18^F]FDG-PET/MRI is being suggested in recent literature as an alternative to WB-MRI and [^18^F]FDG-PET/CT offering the advantage of reduction of administered ionizing radiation since no CT is needed for attenuation correction and ability to lower the [^18^F]FDG dose because of the long acquisition time of the MRI. However, the additional value of PET/MRI is still largely unclear [30, 31].

Several limitations of our study need to be considered. First, since it is undesirable to obtain histopathological evidence of disease presence in all residual lesions, a true gold standard is not available for response assessment in Hodgkin’s lymphoma. It should be taken into consideration that although [^18^F]FDG-PET/CT is the recommended imaging modality in the international guidelines [3], both false-positive and false-negative results do occur [32]. To undertake this lack of a true gold standard, the expert panel used all available data to form the [^18^F]FDG-PET/CT-based reference standard. This use of all clinical, laboratory, follow-up and imaging data caused the reference standard and the index test not to be completely independent from each other. Although this consensus-based method does resemble clinical practice and was used in similar studies before [10, 14], the differences between WB-MRI and the reference standard are probably somewhat underestimated due to this study design.

Second, differences in MRI systems and field strengths were unavoidable due to the multicentre study design, and these differences combined with a long period of inclusion caused quality differences across the study data. This was most visible for the diffusion-weighted images and these differences might have caused bias in the DWI reading and perceptual errors. Furthermore, for this study, only visual assessment of DWI and apparent diffusion coefficient (ADC) images was used rather than the additional value of quantitative ADC measurements in improving the accuracy of WB-MRI. The lack of an optimal ADC cut-off value and the limited accuracy of ADC values for smaller lesions were reasons for the decision to use only DWI assessment only for this study [10, 14, 15, 33]. Concerning the assessment of the DWI, it might have also been beneficial to use a larger variety of *b*-values instead of just b0, b100 and b800. Given that was already shown in the recent literature that the accuracy for distinguishing between benign and malignant lymph nodes is not the same for all *b*-values, adding b200 would probably have been the most beneficial [34].

Third, the intrinsic WB-MRI dataset is probably an overestimation of reality, since reader errors are an unavoidable part of clinical practice. For restaging, no difference was found between WB-MRI including DWI and intrinsic WB-MRI, so the overestimation due to removing reader errors is likely limited. However, for early response assessment, three more patients agreed with the reference standard for intrinsic WB-MRI compared to WB-MRI with DWI; therefore, reader errors were of more influence in early response assessment.

Fourth, in line with the EuroNet protocol [24], only participants with residual disease (uptake above the mediastinal blood pool at [^18^F]FDG-PET) at early response assessment underwent [^18^F]FDG-PET/CT at restaging after completion of therapy, whereas all patients received a restaging WB-MRI. The number of patients that could be included for analysis of restaging data was, therefore, biased and limited to 13 patients, resulting in few residual lesions and limited opportunities for statistical analysis.

Finally, in the EuroNet PHL C1 protocol, determining whether or not the tumours have reduced by at least 50% is also part of early response assessment [24]. We did not implement that criterion in our study, but it could be argued that WB-MRI is viable for determining the percentage of decreased tumour volume and that it could be complementary to [^18^F]FDG-PET/CT in that respect, since the CT is mostly low-dose in early response assessment. Furthermore, as mentioned in the “Methods” section, we used the EuroNet PHL C1 protocol in this study. The current EuroNet PHL study (C2) uses a study protocol that differs from the first, for instance by using a newer Deauville scoring system.

To conclude, the addition of DWI to the WB-MRI protocol in both early response assessment and restaging improved the accuracy in detecting residual disease as compared to an [^18^F]FDG-PET/CT-based reference standard. However, WB-MRI remained discordant in 30% of the patients compared to standard imaging for assessing residual disease presence.

## Supplementary information

ESM 1 (DOCX 23 kb)

## Abbreviations

[^18^F]FDG-PET/CT2: [^18^F]fluoro-2-deoxy-D-glucose positron emission tomography /computed tomography
ABVD: Adriamycin (doxorubicin), bleomycin, vinblastine, dacarbazine
ADC: Apparent diffusion coefficient
CI: Confidence interval
COPDAC: Cyclophosphamide, doxorubicin, prednisone, dacarbazine
CR: Complete response
DECOPDAC: Dacarbazine, etoposide, doxorubicin, cyclophosphamide, vincristine, prednisone
DWI: Diffusion-weighted imaging
ERA: Early response assessment
FN: False negative
FP: False positive
HL: Hodgkin’s lymphoma
IEP: Ifosfamide, etoposide, prednisone
IQR: Interquartile range
MIP: Maximum intensity projection
NA: Not applicable
NPV: Negative predictive value
OEPA: Vincristine, etoposide, prednisone, Adriamycin (doxorubicin)
OPPA: Vincristine, procarbazine, prednisone, Adriamycine
PD: Progressive disease
PPV: Positive predictive value
PR: Partial response
RT: Radiotherapy
SD: Stable disease
TN: True negative
TP: True positive
WB-MRI: Whole-body magnetic resonance imaging
