# Supplementary material for: Whole-body MRI versus an [18F]FDG-PET/CT-based reference standard for early response assessment and restaging of paediatric Hodgkin’s lymphoma: a prospective multicentre study
Source: Eur Radiol. 2021 May 22;31(12):8925–36. doi: 10.1007/s00330-021-08026-1 (PMC8589741; doi:10.1007/s00330-021-08026-1)
Supplement: Supplementary file 1 — (DOCX 23 kb) [file 330_2021_8026_MOESM1_ESM.docx]

**Supplementary material**

The scan protocols were as described before [1].

WB-MRI protocol

WB-MRI was performed using a 1.5-T system (Philips Healthcare or Siemens or GE Medical Systems) or a 3.0-T system (Siemens) and image acquisition took place from the top of the head to the upper thigh (comparable to the scan range commonly applied in [^18^F]FDG-PET/CT imaging). The actual scan range varied slightly according to applied MR hardware and according to height of the patient, but minimally included the upper cervical region to the inguinal/femoral lymph node regions. Choice of appropriate coils for signal reception depended on the available MR system and hardware in each participating centre.

First, coronal whole-body T1-weighted (T1W) and short inversion time inversion recovery (STIR) images were acquired, using the built-in body coil or the whole-body surface coil design for signal reception. Images were acquired under free breathing, except for the stations covering the chest and abdomen, which were acquired using breath holding (T1W) or respiratory triggering (STIR). Second, axial diffusion-weighted images of the head/neck, chest, abdomen, and pelvis were acquired under free breathing.

Data reconstruction was performed on an MRI image analysis workstation (e.g. View Forum, Philips Medical Systems, Best, The Netherlands or Leonardo, Siemens, Erlangen, Germany or Advantage Windows Workstation, GE Medical Systems, Milwaukee). Seamless coronal whole-body T1W and STIR images were created by merging separately acquired stations using software implemented in the standard operating console. Axial diffusion-weighted images were first coronally reformatted with a slice thickness/gap of both 3.5/0mm and 7/0mm, and then merged to create seamless coronal whole-body diffusion-weighted images at b0 s/mm2, b100 s/mm2 and b800 s/mm2. In addition, seamless 3D maximum intensity projection (MIP) reconstructions were created (36 slices x 5 degrees angle in axial plane, rotating around cranio-caudal axis). Whole-body diffusion-weighted images were displayed using greyscale inversion.

The total duration of the examination was about 50-60 minutes, including patient preparation time.

[^18^F]FDG-PET/CT protocol

[^18^F]FDG-PET/CT (Biograph 16 PET-CT or Biograph 40 Truepoint PET-CT, Siemens Healthcare; Gemini TOF PET- CT or Allegro, Philips Healthcare; spatial resolution around 5 mm at full width at half maximum) was performed after at least six hours of fasting. Blood glucose levels were measured and had to be below 11 mmol/L for the procedure to continue. 2 MBq/kg body weight of [^18^F]FDG was administered in every patient. Image acquisition took place 60 minutes after [^18^F]FDG administration.

Low-dose CT scanning was performed with the following settings: 120 kV, 26-30 mAs, 0.8-s tube rotation time, pitch of 1.2, and 1.5-mm slice width (reconstructed to contiguous 5-mm axial slices to match the section thickness of the PET images). After the low-dose CT, PET scanning was performed from mid-thighs to the base of the skull in five- six bed positions, with three minutes per bed position. Low-dose CT was used for attenuation correction of PET data.

PET images were reconstructed using an ordered-subsets expectation maximization algorithm for 14 subsets and four iterations. The image reconstruction matrix was 128 × 128.

The duration of the examination was approximately 90 minutes including the 60 minute waiting time between admission of [^18^F]FDG and the scan.

To minimize the amount of brown fat activation, propranolol was administered one hour before the scan to all patients undergoing an [^18^F]FDG-PET/CT. Children between 10 and 20 kg received 10 mg propranolol whereas children weighing more than 20 kg received 20 mg. After propranolol administration blankets were used to keep the patients warm between administration of propranolol and the scan.

Supplementary table 1. WB-MRI scan parameters

|  | 1.5 T | | | 3.0 T | | |
| --- | --- | --- | --- | --- | --- | --- |
|  | Pulse sequence | | | | | |
|  | T1W TSE | T2W STIR | DWIBS STIR | T1W TSE | T2W STIR | DWIBS STIR |
| Orientation | Coronal | Coronal | Axial/coronal | Coronal | Coronal | Axial/coronal |
| Repetition time (ms) | 518-637 | 2826-6070 | 3200-9754 | 685 | 8289 | 8388 |
| Echo time (ms) | 7-27 | 44-81 | 61-80 | 8 | 70 | 86 |
| Inversion time (ms) | - | 165 | 180 | - | 230 | 220 |
| Slice thickness/ slice gap (mm) | 6/1 | 6/1 | 6/0 | 6/1 | 6/1 | 7/0 |
| Number of slices per station | 30 | 30 | 60 | 30 | 30 | 40 |
| Field of view (mm2) | 530 x 265 | 530 x 265 | 450 x 360 | 549 x 230 | 549 x 230 | 450 x 295 |
| Acquisition matrix | 384 x 256 | 336 x 267 | 128 x 81 | 344 x 292 | 344 x 304 | 128 x 125 |
| B-values (s/mm2) | - | - | 0, 100, 800 | - | - | 0, 800 |
| Number of signals averaged | 1 | 1-2 | 3-5 | 1 | 1 | 1 |
| Respiratory motion compensation | Free breathing, except for thorax and abdomen (breath holding) | Free breathing, except for thorax and abdomen (respiratory triggering) | Free breathing | Free breathing, except for thorax and abdomen (breath holding) | Free breathing, except for thorax and abdomen (breath holding) | Free breathing |

Supplemental table 1: WB-MRI detailed imaging parameters. Parameters vary depending on the system used by the participating centres (Philips, Siemens or GE). *DWIBS, diffusion weighted imaging with background body signal suppression; STIR, short inversion time inversion recovery; T, Tesla; T1W, T1-weighted; T2W, T2-weighted; TSE, turbo spin echo.*

**References**

[1] Spijkers S, Littooij AS, Kwee TC, et al (2020) Whole-body MRI versus an [^18^F]FDG-PET/CT-based reference standard for staging of paediatric Hodgkin lymphoma: a prospective multicentre study. Eur Radiol Online Ahead of Print. doi: 10.1007/s00330-020-07182-0
